# Exploration of candidate serum biomarkers potentially related to the chronic pain condition in Medication–overuse headache

**DOI:** 10.1186/s12883-019-1469-1

**Published:** 2019-10-17

**Authors:** Lanfranco Pellesi, Elisa Bellei, Simona Guerzoni, Maria Michela Cainazzo, Carlo Baraldi, Emanuela Monari, Luigi Alberto Pini

**Affiliations:** 1 Medical Toxicology, Headache and Drug Abuse Centre, University of Modena and Reggio Emilia, Modena, Italy; 2 Department of Diagnostic Medicine, Clinic and Public Health, Proteomic Lab, University of Modena and Reggio Emilia, Modena, Italy; 3 Center for Neuroscience and Neurotechnology, University of Modena and Reggio Emilia, Modena, Italy

**Keywords:** Biomarker, Allodynia, L-PGDS, VDBP, APOE, APOA1, ELISA

## Abstract

**Background:**

Medication Overuse Headache (MOH) is a prevalent and disabling disorder resulting from the overuse of analgesic drugs, triptans or other acute headache medications. In previous proteomic studies, several proteins have been found at high concentrations in the urine of MOH patients and in the serum of rats with neuropathic pain. The aim of this study was to compare the serum levels of lipocalin-type Prostaglandin D2 synthase (L-PGDS), Vitamin D-binding protein (VDBP), apolipoprotein E (APOE) and apolipoprotein A1 (APOA1) in MOH patients and healthy individuals, further exploring their relationship with cutaneous pain thresholds (CPTs) in the territories innervated by the trigeminal nerve.

**Methods:**

Sixty-nine MOH patients and 42 age- and sex-matched healthy volunteers were enrolled in the study. Von Frey-like filaments were applied to the skin territories innervated by the trigeminal nerve, to determine the CPTs. L-PGDS, VDBP, APOE and APOA1 were quantified in the serum by Enzyme-linked Immunosorbent Assay (ELISA). Clinical and laboratory data were collected. Comparisons between MOH patients and healthy individuals were performed using independent t test or χ^2^ test. To correlate serum proteins with CPTs, Pearson correlation coefficient or Spearman’s rank correlation coefficient were used.

**Results:**

CPTs were lower among MOH patients. L-PGDS, VDBP and APOE had significantly different serum concentrations between groups (*p* < 0.01), but no correlation was found with CPTs. APOA1 serum concentrations did not differ between patients and healthy individuals.

**Conclusions:**

L-PGDS, VDBP and APOE had abnormal serum levels in MOH patients, confirming their alteration in some conditions of chronic headache and neuropathic pain. However, they had no relationship with CPTs. The in-depth study of serum proteins represents a promising approach for a better understanding of MOH, as well as the detection of candidate biomarkers for chronic headache or the risks associated with overuse medications.

## Background

Chronic pain (CP) is a major health care problem. It affects approximately 20% of the European population [1], and gives considerable costs to the society, more than heart diseases, cancer and diabetes put together [2]. CP is a persistent and disabling pain over a long period, starting from tissue injuries or inflammation, or from lesions of the nervous system [3]. The maintenance of pain often aggravates the patient’s symptoms, originating the phenomenon of central sensitization, which manifests itself as pain hypersensitivity, allodynia and hyperalgesia [4, 5]. Currently, CP treatment has limited effect because of poor understanding of the mechanisms that lead to the initiation and maintenance; the complete elimination of pain is rarely obtainable. Important reasons for this failure are the high variability of CP manifestations, as well as the absence of distinctive and measurable biological traits.

Headaches are prevalent among chronic painful disorders. It is estimated that up to 5% of the general population suffers from chronic headache, defined as headache occurrence ≥15 days per month [6]. Chronic forms differ from the episodic forms not only in frequency, but also in the lack of effectiveness of most therapies, more medication overuse and lower quality of life [7]. The almost daily intake of acute headache medications can paradoxically worsen the chronic symptoms, leading to a secondary headache named medication-overuse headache (MOH) [8]. MOH may complicate the majority of primary headaches and it can be caused by several acute headache medications, including migraine-specific (triptans and ergotamine) and nonspecific (nonsteroidal anti-inflammatory drugs or NSAIDs, paracetamol, opioids and combinations of analgesics or mixture) drugs. Every class of overused drugs may have a precise causative role, but the repeated intake of the drug does not seem enough to generate MOH. When the acute headache medication is taken regularly for non-headache indications, people without a history of headache do not develop MOH [9, 10]. Thus, the association between an individual predisposition and the effects of the headache medications are probably crucial in its outcome [11]. At present, there are no recognisable pathological traits associated with MOH. The only proposed was cutaneous allodynia (CA) [12], defined as pain resulting from an innocuous stimulus to normal skin.

Recently, several urinary proteins have been found at high levels in MOH patients [13, 14]. One of the most interesting was lipocalin-type prostaglandin D2 synthase (L-PGDS). The enzyme is responsible for the biosynthesis of prostaglandin D2 (PGD2), the most abundant prostanoid produced in the central nervous system (CNS) [15]. PGD2 is involved in a variety of physiological functions, such as sleep induction, body temperature regulation, vasodilation and pain responses, including allodynia [16–19]. Although the role of L-PGDS is not completely defined, the entire prostaglandin system has a central position in pain and central sensitization, resulting in induction of hyperalgesia and CA [20–25]. These findings have been deepened into an animal model of neuropathic pain, revealing an increased expression of L-PGDS as well. Several other proteins were identified in the rat serum 5 weeks after the chronic constriction of sciatic nerve, including Vitamin D-binding protein (VDBP), apolipoprotein E (APOE) and apolipoprotein A1 (APOA1) [26]. Nonetheless, quantitative Polymerase Chain Reaction (qPCR) analysis showed that their transcript levels were overexpressed in the lumbar spinal cord (origin of sciatic nerve), and not in the striatum (an unrelated brain region) [26].

Assuming that some pathophysiological processes may be shared between MOH and neuropathic pain, the primary objective of this study was to quantify and compare the serum levels of L-PGDS, VDBP, APOE and APOA1, as well as the cutaneous pain thresholds (CPTs) in the territories innervated by the trigeminal nerve in MOH patients and healthy individuals as control group. Secondarily, serum levels of proteins have been correlated with CPTs in MOH patients.

## Methods

### Population of the study

Between September 2016 and January 2018, 69 consecutive MOH inpatients, referring to the Headache and Drug Abuse Centre at the University of Modena and Reggio Emilia, were enrolled in the study. All patients were diagnosed by trained physicians, according to the International criteria [8]. After signing written informed consent, the collection of demographic data, the measurement of blood pressure and experimental procedures were completed on admittance, before withdrawal treatment was initiated. Patients were tested in the morning, before taking any therapy. A trained investigator performed the CPT tests. The blood and urine routine laboratory tests were performed on the second day of hospitalization, fasting from midnight. For each patient, the subsequent parameters were analysed: (1) prevalent acute headache medication used, classified in triptans, NSAIDs and mixtures (2) daily drug intake (DDI), mean number of acute headache medications consumed every day; (3) years of chronification (CHR), time elapsed since the beginning of chronic symptoms. Prevalent acute headache medication used and DDI were referred to a 3-month period before testing.

Forty-two age- and sex-matched healthy volunteers were enrolled as controls. They were recruited from the hospital’s medical staff and their relatives. On the experimental day, they also performed blood tests for renal function. Only healthy subjects without migraine and any headache disorders were included in the study, except for tension-type headache for no more than 3 days per month. The daily intake of medicines and the assumption of acute pain medications more than five times in the last month or in the previous 48 h of the experimental procedures were considered exclusion criteria for the study. For all study participants, exclusion criteria were: headache on admittance to the centre, altered results in routine laboratory tests, other non-cephalic chronic painful conditions, and autoimmune, hepatic, renal, oncological, cardiovascular, psychiatric or neurological relevant pathologies.

### Serum samples collection and storage

Venous blood was collected in vacutainer tubes and allowed to clot at room temperature for 1 h. Serum was obtained by centrifugation at 2000 x g for 10 min at 4 °C; a mixture of protease inhibitors (Sigma-Aldrich) was added to prevent protein degradation and alteration. Samples were divided into aliquots and stored at − 80 °C until analysis.

### ELISA test

Globally, 111 serum samples, which were comprised of 69 MOH patients and 42 healthy individuals, were analysed by ELISA test. Especially, the expression level of the following proteins was evaluated: L-PGDS (human Prostaglandin D Synthase, Lipocalin-type, ELISA kit, BioVendor, Brno, CZ), VDBP (human Vitamin D-binding protein, ELISA kit, Cusabio Biotech, USA), APOE (human apolipoprotein E, Apo-E, ELISA kit, Cusabio Biotech, USA) and APOA1 (human apolipoprotein A1, Apo-A1, ELISA kit, Cusabio Biotech, USA). The assays were performed according to the manufacturer’s protocols and instructions.

L-PGDS and APOA1 assays employ the quantitative sandwich enzyme immunoassay technique, while the VDBP and APOE kits use the competitive inhibition enzyme immunoassay method. Briefly, in the first case, diluted serum samples, standards and blanks were dispensed in the microplate wells containing immobilized pre-coated specific antibody for L-PGDS and APOA1, respectively. After 1 h incubation, a horseradish peroxidase (HRP)-conjugated antibody specific for L-PGDS and APOA1 was added in each well of the plate, which was incubated further for 1 h. Following a wash to remove any unbound reagent, a substrate solution was added and allowed to react with HRP-conjugate. A blue colour proportional to the amount of sample protein bound in the initial step developed. The reaction was interrupted by an acid solution, which lead to a yellow product. The absorbance was measured at λ 450 nm in a microplate reader (Multiscan FC, Thermo Scientific, USA). Protein concentrations were calculated from a standard curve generated by the protein stock solution furnished with the kit.

VDBP and APOE kits used a microtiter plate pre-coated with the respective proteins. Standards and samples (50 μL) were added to the plate wells, together with the same amount of HRP-conjugated antibody specific for the protein, and incubated at 37 °C. This step was followed by aspiration and washing repeatedly for five times before adding 90 μL of tetramethylbenzidine (TMB) substrate solution to each well (in this case, the colour developed in an opposite way to the amount of protein present in the serum sample). After 10 min incubation at 37 °C in the dark, the reaction was interrupted by adding 50 μL of stop solution and the colour intensity was measured within 5 min at λ 450 nm.

### Cutaneous pain thresholds

All the CPTs assessments were performed in absence of a visible headache attack. Calibrated von Frey-like filaments (Touch-Test® Sensory Evaluators filaments, North-Coast Medical Inc., CA) were applied sequentially in scheduled areas, in increasing order for 2 seach to determine the basal CPTs, asking the patient when the touch became a painful or very uncomfortable sting. Assessments were repeated three times and the filaments were used in each location in the following sequence, representing the increased strengths measured in grams (e.g. 0.008; 0.02; 0.04; 0.07; 0.16; 0.4; 0.6; 1; 1.4; 2; 4; 6; 8; 10; 15; 26; 60; 100; 180 and 300 g). The test investigated three cutaneous areas: temple, cheekbone and chin areas (exploring first, second and third division of the trigeminal nerve, respectively), assessed bilaterally in random order. Landmarks definitions were: temple, the skin over the pars orbitalis of frontal and sphenoid bones; cheekbone, the skin over periorbital area of zygomatic bone; chin, the skin of the chin, including the innervation territories of the lower alveolar nerves.

### Statistical analysis

Quantitative variables were expressed as a mean ± standard deviation. Qualitative variables were expressed as a percentage. Comparison of quantitative variables between MOH patients and healthy individuals was performed using independent *t* test, whereas comparison of qualitative variables was performed using χ^2^. All *p*-values are two-sided, with a level of significance of 1%. To correlate serum L-PGDS, VDBP, APOE and APOA1 with CPTs, data were analysed with the Pearson correlation coefficient or, if one of the two variables was not normally distributed, Spearman’s rank correlation coefficient was used. R software (version 3.4.3) was used to perform statistical analysis. The level of significance for correlation tests was set at 5%.

## Results

### Population characteristics

We enrolled 69 MOH patients, aged between 27 and 74 years (mean = 50.2), most of them were females. In more than 90% of cases, the pre-existing headache disorder was migraine. Tension-type headache was diagnosed as pre-existing headache disorder in three patients, while post-traumatic headache in one patient. On admittance, headache was daily or almost daily for all patients, for not less than 3 months. They assumed about two acute headache medications a day (mean = 1.8, range = 0.33–9), and had been suffering of more than 10 years of chronic headache (mean = 12.1, range = 0.33–35). According to the prevalent use of acute headache medications, the majority assumed triptans (*n* = 48; 70%), fewer patients were taking NSAIDs (*n* = 10; 14%) or mixture drugs (*n* = 11; 16%). The latter consisted of a drug containing indomethacin, caffeine and prochlorperazine in 90% of cases, the remaining ones were codeine plus paracetamol. Blood and urine laboratory tests showed no alterations in any parameter, including liver and kidney function. The control group of 42 healthy individuals was an equally female-dominated group, aged between 25 and 76 years (mean = 47.0). Blood tests performed did not reveal abnormal renal function. Compared to healthy subjects, MOH patients had higher Zung Self-Rating Depression Scale (ZUNG-D) scores and less prevalent alcohol consumption. No other differences were observed between the two groups. Data and statistical comparisons are summarized in Table 1.

**Table 1:** Demographic, clinical and laboratory data of MOH patients and healthy individuals

	MOH patients (*n* = 69)	Controls (*n* = 42)
Age, years	50.2 ± 9.2	47.0 ± 13.8
Sex, female	90%	88%
BMI, kg/m^2^	23.7 ± 4.5	23.7 ± 3.8
Coffee	84%	88%
Alcohol	22%*	76%
Cigarettes	9%	12%
SBP, mmHg	125.9 ± 12.4	122.1 ± 13.0
DBP, mmHg	79.3 ± 8.2	76.8 ± 8.5
DDI	1.8 ± 2.1	–
CHR	12.1 ± 10.3	–
ZUNG-D	44.3 ± 9.5**	32.3 ± 7.7
LDQ	12.1 ± 7.4	–
Creatinine, mg/dl	0.76 ± 0.12	0.79 ± 0.12
eGFR	> 60 ml/min	> 60 ml/min
Urea nitrogen, mg/dl	29.4 ± 7.4	29.6 ± 8.5

Data are expressed as mean ± SD, or in percentage

*BMI* Body Mass Index; *SBP* systolic blood pressure; *DBP* diastolic blood pressure; *DDI* daily drug doses; *CHR* years of chronification; *ZUNG-D* Zung Self-Rating Depression Scale; *LDQ* Leeds Dependence Questionnaire; *eGFR* estimated Glomerular Filtration Rate (CKD-EPI Formula)

Statistical significance was evaluated using χ^2^ test (*p < 0.01) or independent *t* test (**p < 0.01). Coffee, alcohol and cigarettes users are expressed as a percentage of occasional and daily users

### ELISA test

**Fig. 1:**
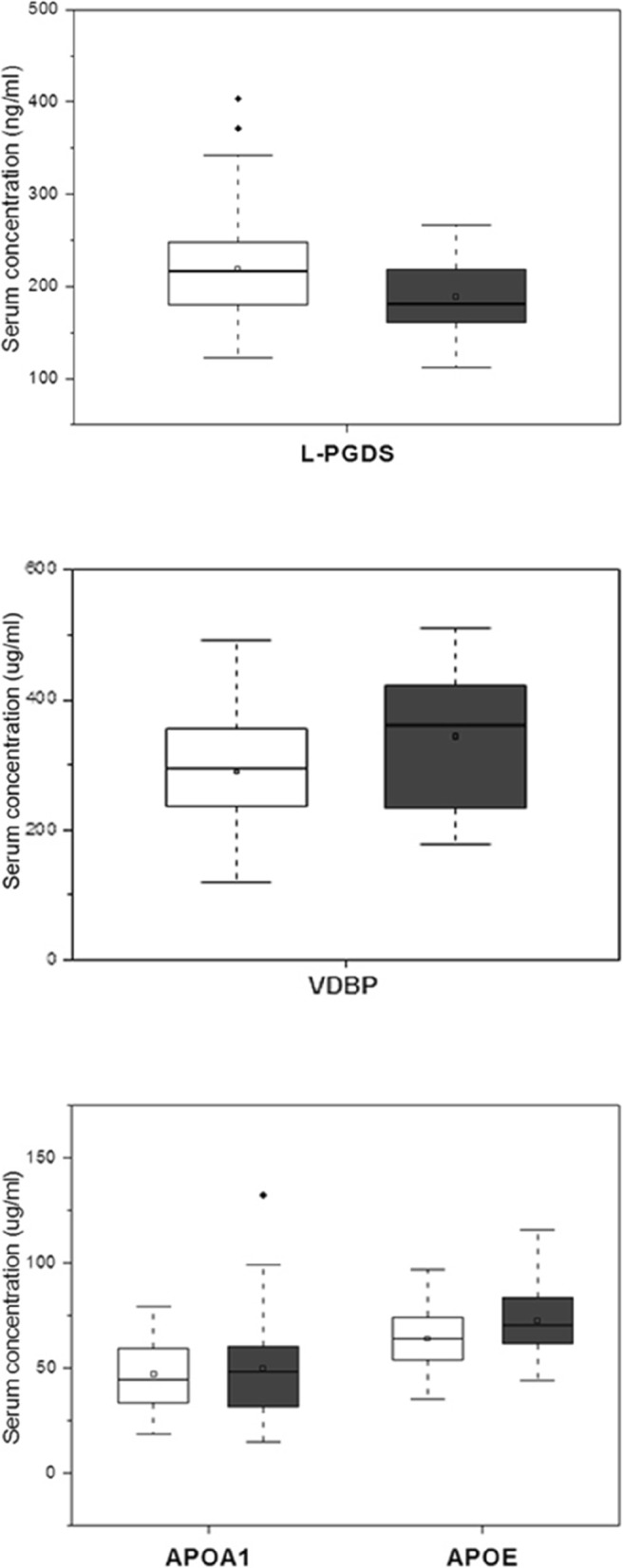
Box plots of serum concentrations of L-PGDS, VDBP and apolipoproteins in MOH patients (white) and healthy individuals (grey)

The level of L-PGDS and APOE was estimated in serum samples collected from 69 MOH patients and 42 healthy participants. Regarding L-PGDS, a significant increase was observed in patients, compared to healthy individuals (219.2 ng/ml vs. 188.7 ng/ml, *p* < 0.01). On the other hand, the serum concentration of APOE was significantly higher in healthy individuals (72.5 μg/ml vs. 63.9 μg/ml, *p* < 0.01). The level of serum VDBP was estimated in a lower number of samples, i.e., in 57 MOH patients and 31 healthy individuals. This was due to technical problems experienced during the laboratory practice. The control group showed significantly higher levels of serum VDBP (343.7 μg/ml vs. 289.5 μg/ml, *p* < 0.01). Regarding serum APOA1, no differences were found between 57 MOH patients and 30 healthy participants (49.8 μg/ml vs. 46.9 μg/ml, *p* = 0.5466). Dosages have been performed in fewer samples, due to technical laboratory biases. According to the prevalent acute headache medication used, the concentration of serum proteins was relatively stable. Data regarding the serum concentrations of proteins are summarized in Table 2 and Table 3, as well as better displayed in Fig. 1.

**Table 2:** CPT values and serum proteins levels in healthy individuals and MOH patients

	Controls	MOH patients	*P*-value
Mean CPT (g)	96.0 ± 41.0	46.4 ± 27.7	< 0.00001^**^
L-PGDS (ng/ml)	188.7 ± 39.2	219.2 ± 58.2	0.0038^**^
VDBP (μg/ml)	343.7 ± 95.3	289.5 ± 88.7	0.0058^**^
APOE (μg/ml)	72.5 ± 17.3	63.9 ± 13.3	0.0042^**^
APOA1 (μg/ml)	46.9 ± 17.5	49.8 ± 23.7	0.546^NS^

CPT values and serum protein concentrations are expressed as mean ± standard deviation (SD).Independent *t* test (patients vs. controls): statistical significance ^**^: *p* < 0.01, ^NS^: not significant

**Table 3:** Serum proteins levels in MOH patients, according to the prevalent acute headache medication

	Triptans	NSAIDs	Mixtures
L-PGDS (ng/ml)	209.4 ± 57.5	214.9 ± 53.5	266.0 ± 44.6
VDBP (μg/ml)	296.0 ± 86.0	262.3 ± 67.4	287.8 ± 124.0
APOE (μg/ml)	65.6 ± 13.2	55.6 ± 10.7	64.2 ± 14.2
APOA1 (μg/ml)	46.6 ± 20.3	60.5 ± 23.0	53.3 ± 33.3

Serum protein concentrations are expressed as mean ± standard deviation (SD)

### Cutaneous pain thresholds

**Fig. 2:**
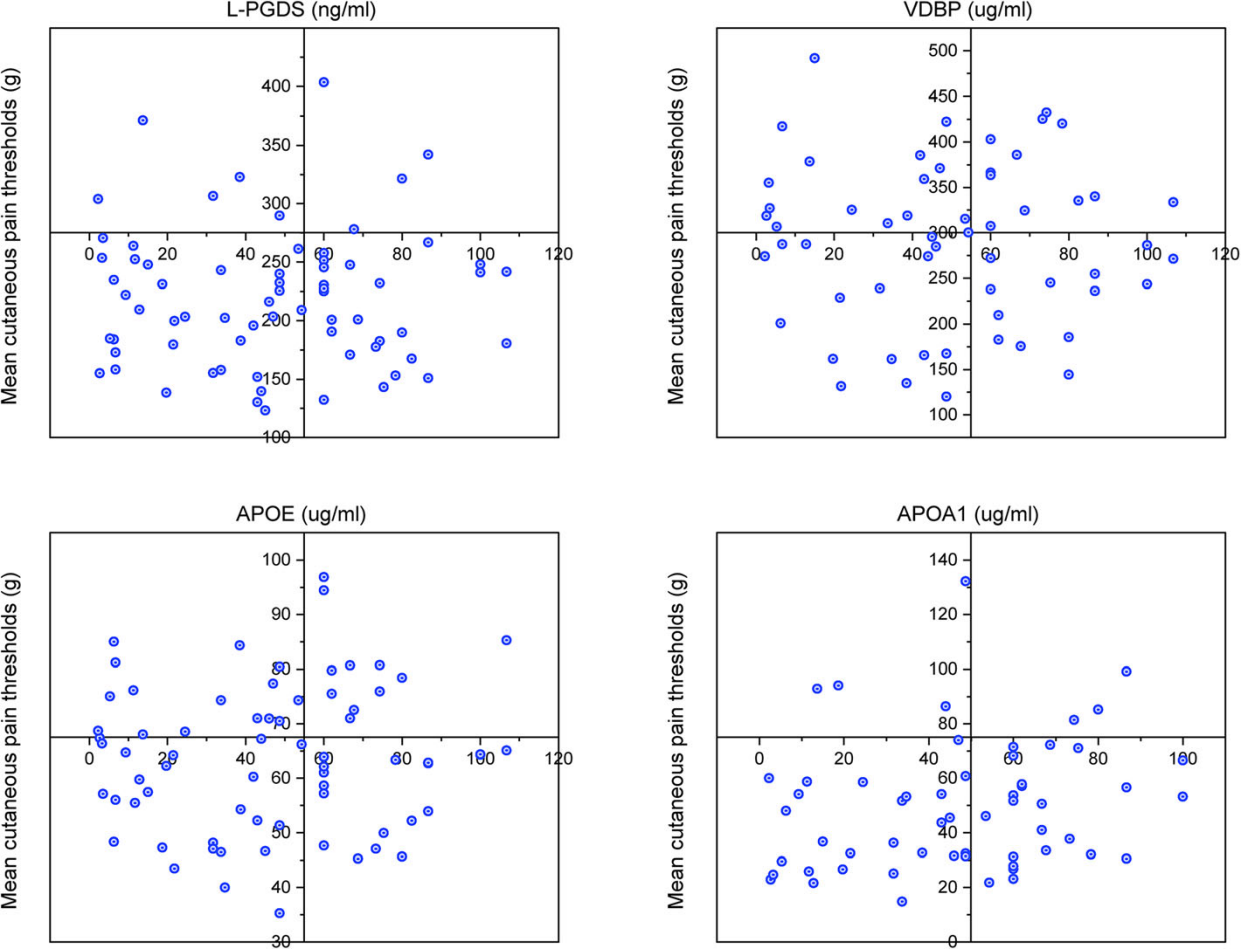
Relationship between mean CPT values and serum concentration of target proteins

According to the absence of any significant difference between right and left sides and the three trigeminal branches, CPTs are presented as an overall average. Compared to healthy participants, mean CPT values in the three tested areas (temples, cheekbone and chin) were significantly lower in MOH patients (46.4 g vs. 96.0 g, *p* < 0.01). No statistical differences were found between males and females. Data and statistical comparisons are described in Table 2. When the mean CPTs values are compared with the serum concentration of the serum proteins, no relationship is detectable for L-PGDS (Spearman’s rank = − 0.001; *p* = 0.9921), VDBP (Spearman’s rank = − 0.147; *p* = 0.4301), APOE (Spearman’s rank = 0.053; *p* = 0.6667) and APOA1 (Spearman’s rank = 0.25769; *p* = 0.05296). Graphic correlations are displayed in Fig. 2.

## Discussion

The main findings of this study concern the serum differences of L-PGDS, VDBP and APOE in MOH patients, compared to healthy individuals. These discrepancies were not one-sided, as L-PGDS was identified at high concentrations, while VDBP and APOE were deficient. Our group of patients with MOH was nearly aligned with previous case studies [27], confirming to have a higher prevalence of CA than healthy individuals [12, 28]. Of note, the intake of opioids and drugs containing barbiturates was lower; this might be due to population differences [29] or to the study selection criteria, which excluded patients with other painful syndromes and comorbidities. These assumptions were ideal to correlate the serum concentration of L-PGDS, VDBP and APOE to the pain thresholds measured on the skin of the face. However, none of these proteins (including APOA1) showed any relationship with CPTs.L-PGDS was already found at unusually high concentrations in urine of MOH patients [13, 14]. The enzyme is highly expressed in leptomeninges [30] and is physiologically eliminated via the kidney. It is of interest for headaches and migraine, as it synthesizes PGD2, a strong vasodilator of the human middle cerebral artery [25], as well as a stimulus for the release of calcitonin gene-related peptide (CGRP) from trigeminal neurons [31]. Alterations in concentration of L-PGDS have also been discussed in other neurological diseases, including cerebral infarction, multiple sclerosis and schizophrenia [32, 33]. Anyway, its high serum concentration remains unexplained, did not exhibiting any correlation with CA. Another likely explanation behind its high concentration is related to the frequent intake of pain relievers. The common and prolonged assumption of acute headache medications is not uniquely associated with worsening headache, but also to stomach bleeding, liver and kidney damage. NSAIDs use was associated with a 3-fold greater risk for acute renal failure compared with non-NSAIDs use; this risk was dose-dependent and increased with long-term use [34]. About triptans, their potential kidney injury is less known, although some cases of renal infarction have been reported after prolonged use [35]. By adding that L-PGDS accumulates in the serum of patients with impaired renal function, especially in dialysis patients (ca. 100-fold), and it has been proposed in the past as a diagnostic marker for early renal injury [36, 37], the renal impairment may be a valid argument for its elevated levels. However, the renal function indicators in the blood of MOH patients were not different from those of healthy volunteers.

Serum concentrations of VDBP and APOE were higher in healthy individuals. VDBP is primarily a carrier and transporter of vitamin D in the circulation. It also is a component of the extracellular actin-scavenger system, comprising gelsolin; specifically, VDBP binds and facilitates clearance of monomeric actin, released upon injury [38]. Furthermore, VDBP works as a macrophage-activating factor (MAF), initiating macrophage activity [39]. The current result contradicts the findings in rats, where the serum level of VDBP was elevated up to 5 weeks, after the chronic constriction injury of the sciatic nerve [26]. However, this agrees with other human studies which investigated its role in chronic painful conditions. With a methodological approach like the one we adopted, serum VDBP was identified among the downregulated spots in carpal tunnel syndrome patients, compared to healthy individuals [40]. Moreover, a Japanese family study identified a possible dysfunction of VDBP in migraineurs [41]. It has been assumed that a lower binding of the VDBP to monocyte chemoattractant protein-1 (MCP-1), a member of the monocyte chemoattractant protein family, may be involved in the development of migraine pain. Elevated CSF levels of MCP-1 were already reported in migraine patients [42] and the chemokine can also trigger CGRP release [43]. Regarding vitamin D, the natural ligand of VDBP, more data are available. It has been associated with the aetiology and persistence of painful conditions [44], and its supplementation can relieve pain in most patients with a vitamin D deficiency [45]. Interestingly, vitamin D inadequacy has been linked to higher doses of analgesics among patients with chronic musculoskeletal pain [46], In migraine patients, serum vitamin D and Vitamin D receptor levels were found to be lower than in controls, whereas serum VDBP levels were similar between the two groups [47]. In this study, Vitamin D was not dosed. In the future, interesting advancements in MOH could concern the dosage of vitamin D and MCP-1, together with a re-evaluation of the VDBP protein.

More concealed is the association between APOE and pain regulation processes [48]. It transports cholesterol into the blood and has many essential functions in the brain, including synaptogenesis, regulating synaptic transmission and promoting nerve regeneration after a nerve injury [49]. Despite intriguing reasons that favour the association between APOE and pain, few human studies were conducted to date. In relation to headache conditions, APOE-2 allele has been identified as a risk factor for migraine, whereas APOE ε4 allele is positively related to headache, including migraine and tension-type headache [50, 51]. Yet, further research is required for the reason behind the low serum concentration of APOE in patients with MOH.

The study has some limitations. First, our patients were mainly represented by women, but an evaluation of the serum proteins in the light of the hormonal state is lacking. And, the association between these proteins and the overuse medication has not been studied exhaustively. The association between proteins and pain might be missing because the protein markers are possibly de-regulated from the pain state. Presumably, such alterations are not directly related to the central pain sensitization, rather to other aspects of chronic headache or the excessive intake of acute headache medications, e.g. the decrease of VDBP and APOE could indicate liver stress due to excessive medication intake. Anyway, the liver function of MOH patients evaluated at the admission to the hospital were not abnormal. Alternatively, these proteins might actually be related to some aspect of the chronic pain condition, but CPTs might function only as a proxy for the medication overuse. In order to understand whether medication overuse affect the serum proteins, it would be interesting to study how these drugs change in the light of improvements due to medication withdrawal or prophylactic therapies. Several biomarkers were already investigated in MOH, including genetic polymorphisms and neuroimaging abnormalities [52], however the exploration of serum biomarkers remain more attractive and desirable, having the appeal of not be affected by the patient’s hydration status and urine flow rate, as well as being easily measurable and suitable for follow-up.

## Conclusions

In conclusion, this study has demonstrated differences in the concentration of distinct serum proteins between MOH patients and healthy individuals. However, none of them appear to be involved in facial allodynia, one of the most distinctive aspect of MOH patients. Presumably, such alterations are not related to the central sensitization, rather to the excessive intake of acute headache medications. It would now be interesting to study these markers and their role in medication overuse, correlating their progress after a medication withdrawal or the start of prophylaxys therapy. In the future, it would be also of interest to explore the levels of drugs (e.g., triptans, NSAIDs, paracetamol, …) and correlate them with the protein markers and/or other clinical aspects of MOH.

## Data Availability

All relevant data are within the paper. The datasets used and/or analysed during the current study are available from the corresponding author on reasonable request.

## Abbreviations

APOA1: Apolipoprotein A1
APOE: Apolipoprotein E
CA: Cutaneous allodynia
CGRP: Calcitonin gene-related peptide
CHR: Years of chronification
CNS: Central nervous system
CP: Chronic pain
CPTs: Cutaneous pain thresholds
CZ: Czech Republic
DDI: Daily drug intake
ELISA: Enzyme-linked Immunosorbent Assay
HRP: Horseradish peroxidase
L-PGDS: Lipocalin-type Prostaglandin D2 synthase
MAF: Macrophage-activating factor
MCP-1: Monocyte chemoattractant protein-1
MOH: Medication Overuse Headache
NSAIDs: Nonsteroidal anti-inflammatory drugs
PGD2: Prostaglandin D2
qPCR: Quantitative Polymerase Chain Reaction
TMB: Tetramethylbenzidine
USA: United States
VDBP: Vitamin D-binding protein
ZUNG-D: Zung Self-Rating Depression Scale
